# *Pseudomonas otitidis* bacteraemia in a patient with COPD and recurrent pneumonia: case report and literature review

**DOI:** 10.1186/s12879-021-06569-8

**Published:** 2021-08-25

**Authors:** Adriana L. Caixinha, Alexandros N. Valsamidis, Ming Chen, Mats Lindberg

**Affiliations:** 1 Emergency Department, University Hospital of Southern Denmark, Kresten Philipsens Vej 15, 6200 Aabenraa, Denmark; 2 Department of General Surgery, University Hospital of Southern Denmark, Aabenraa, Denmark; 3 Department of Microbiology, University Hospital of Southern Denmark, Soenderborg, Denmark

**Keywords:** *Pseudomonas otitidis*, Bacteraemia, COPD, Pneumonia

## Abstract

**Background:**

*Pseudomonas otitidis* is a novel species of *Pseudomonas* bacteria that has been isolated from patients with otic infections.

**Case presentation:**

In this report, we describe a case of a 59-year-old known with moderate chronic obstructive pulmonary disease with bronchiectasis and recurrent pneumonia where blood cultures revealed the growth of *P. otitidis*.

**Conclusions:**

This case describes the first report of bacteraemia to *P. otitidis* and raises questions regarding the misdiagnosis and underestimation of the incidence of infections caused by this novel pathogen.

## Background

*Pseudomonas otitidis* is a gram-negative rod-shaped bacterium first described in 2002 and reported exclusively as an otic infectious agent [1]. In this article, we present the unique case of bacteraemia due to *P. otitidis.* The patient suffered from chronic obstructive pulmonary disease and recurrent pneumonia.

## Case presentation

A 59-year-old man with a medical history of chronic obstructive pulmonary disease (COPD), epilepsy, abdominal aortic aneurysm, previous right parieto-occipital ischemic stroke and arterial small vessel disease was admitted to the emergency department with anorexia, severe weight loss and worsened general condition.

Physical examination revealed a cachectic man with a BMI of 15 kg/m^2^. Body temperature was 37.7 °C. Oxygen saturation was 93% with FiO2 of 25%, and respiration rate was 20 cycles per min (cpm). Blood pressure was 105/44 mmHg, and heart rate was 81 beats per min (bpm).

Blood tests revealed an elevated C-reactive protein (CRP) of 150 mg/L, a slight leucocytosis of 10.9 10^9^/L, macrocytic anaemia with a haemoglobin of 6.2 mmol/L, multiple vitamin and mineral deficiencies (folic acid, iron, zinc, magnesium and vitamin D) and normoglycaemia. Blood and urine cultures were negative.

On chest X-ray, there were signs of chronic lung fibrosis and emphysema, but no visible infiltrates, bronchiectasis or pleural effusion. An abdominothoracic computed tomography (CT) revealed a right upper lobe infiltrate, bilateral bronchiectasis and enlarged mediastinal lymph nodes without signs of malignant disease.

Treatment with piperacillin-tazobactam was initiated along with a diet plan and vitamin supplements. Four days after admission, the patient was discharged against medical advice. At the time of discharge, the patient had a declining, though still elevated, CRP of 80 mg/L, normalised leucocytes of 6,37 10^9^/L and stationary haemoglobin of 6,3 mmol/L. He was prescribed amoxicillin-clavulanic acid for the next 10 days and was referred to his general practitioner for follow-up blood tests. A thoracic CT scan was planned 2 months later.

Approximately 2 weeks after the discharge, the patient was readmitted. He reported fever, productive cough and progressive dyspnoea. He assured us that he had taken his medication as prescribed at discharge.

Physical examination revealed a body temperature of 39.5 °C, respiration rate of 28 cpm, oxygen saturation of 90% (FiO2 21%), pulse rate of 131 bpm and blood pressure of 143/76 mmHg. Arterial blood gas analysis showed hypoxemia and respiratory alkalosis (pH 7.49, pO2 8.1 kPa, pCO2 3.6 kPa and hydrogen carbonate 22.6 mmol/L). Arterial lactate was normal.

Laboratory findings reported a CRP of 168 mg/L, leucocytosis of 17.7 10^9^/L with neutrophilia of 15.7 10^9^/L and lymphopenia of 0.72 10^9^/L and normoglycaemia. Renal and hepatic parameters were normal. Once again, the chest X-ray showed emphysema and fibrosis without visible infiltrates or pleural effusion. The urine test strip had no traces of leucocytes, nitrite, blood or protein. The SARS-CoV-2 PCR swab test, the *Pneumococcal* and *Legionella* urinary tests and urine cultures were all negative.

As there was fever with an obvious respiratory focus but no bacteriological diagnosis, blood cultures were obtained. After 33 h of incubation, gram-negative rods were found in one of the blood cultures. Using Matrix-assisted laser desorption/ionisation time-of-flight mass spectrometry (MALDI-TOF MS), the microorganism was identified as *P. otitidis*. The isolate was sent to the Danish State Serum Institute for identification and confirmed as *P. otitidis*, using 16 S rRNA sequence analysis. The bacterium was sensitive for piperacillin-tazobactam, gentamicin and ciprofloxacin. In collaboration with the Department of Clinical Microbiology, a 7-day-treatment of intravenous piperacillin-tazobactam and gentamicin was initiated.

An otorhinolaryngologic examination was performed to exclude asymptomatic otitis. Neither otoscopic examination nor flexible laryngoscopy showed any signs of pathology.

The patient completed 7 days treatment with piperacillin-tazobactam and gentamicin and was discharged clinically well. On discharge, the patient had a CRP of 20 mg/L and leucocytes of 10,7 10^9^/L, and a prescription for oral amoxicillin-clavulanic acid for 10 days. He was referred to the general practitioner for follow-up blood tests. Based on the patient’s symptoms at admittance and the lung infiltrates seen on the recent thoracic CT scan, the discharge diagnosis was pneumonia.

Approximately 1 month later, the patient was readmitted with the same symptoms. A new thoracic CT scan showed bilateral pneumonia and bilateral bronchiectasis. No positive blood cultures were obtained. The patient underwent a fibrobronchoscopy with bronchoalveolar lavage, which revealed normal cytology and no bacterial growth. After completing a new series of IV piperacillin-tazobactam, the patient was discharged clinically well.

## Discussion and Conclusion

In 2002, a novel *Pseudomonas* species was identified from clinical specimens of infected human ears. Biochemical characterisation and genetic analyses of isolates from patients with acute otitis externa, acute otitis media with otorrhoea or chronic suppurative otitis media led to the description of this novel bacterium. It was found to be closely related to *P. aeruginosa*, and the name *P. otitidis* was proposed [1, 2].

*Pseudomonas otitidis* was initially reported exclusively as an otic infectious agent, but in 2016, the first report of non-otic *P. otitidis* infection was published. In this case report, the bacterium was isolated from a wound culture in a patient with necrotising fasciitis and the peritoneal fluid of a patient with pan-peritonitis. Both patients were immunocompromised [3].

To the best of our knowledge, this is the first report of *P. otitidis* bacteraemia. The focus of *P. otitidis* infection was presumably the lung or the airways, and the clinical presentation was pneumonia in a frail patient with moderate COPD and bronchiectasis. Airway colonisation with the closely related bacteria *P. aeruginosa* is a well-known problem in patients with cystic fibrosis or bronchiectasis but is also reported frequently in severe COPD [4]. In a Danish case series of 113 non-cystic fibrosis patients with *P. aeruginosa*-positive airway samples, the majority suffered from COPD [5].

Our patient illustrates the difficulties in eradicating *Pseudomonas* species from colonised airways. In an observational study of 3193 patients with community-acquired pneumonia, the prevalence of *P. aeruginosa* overall was 4.2%, but among patients with prior infection/colonisation due to *P. aeruginosa* and tracheostomy, bronchiectasis or severe COPD, the prevalence was 67% [6].

Selection of the most appropriate oral antibiotic regimen at discharge is a significant challenge in treating pneumonia caused by the *Pseudomonas* species. Our patient was twice prescribed amoxicillin-clavulanic acid, which was not the optimal choice as *Pseudomonas* species almost always show resistance to amoxicillin. In Denmark, fluoroquinolones are the only class of oral antibiotics effective against the *Pseudomonas* species, but a combination treatment with at least two antibiotics is recommended to avoid the development of antibiotic resistance. In order to eradicate *Pseudomonas*, prolonged parenteral antibiotic treatment is therefore necessary.

Gram-negative rods, including *Pseudomonas* species, are routinely cultured in CHROMagar™ Orientation plate at 35 ºC. Before the introduction of MALDI-TOF MS to clinical microbiology laboratories, identification of the bacteria was performed by a phenotypic expert system, VITEK 2 system (BioMérieux, France). *P. otitidis* is challenging to identify with these phenotypic tests as its phenotype is similar to *P. aeruginosa*. Given their similarity, we believe that the rarity of the detection of *P. otitidis* may partly be due to misidentification as *P. aeruginosa*. Using advanced diagnostic methods such as MALDI-TOF MS and gene sequencing, correct identification can readily be obtained, but such methods have only been used routinely during the last decade.

To the best of our knowledge, this is the first reported case of *P. otitidis* bacteraemia. The patient suffered from moderate COPD with bronchiectasis and recurrent pneumonia. Treatment with intravenous piperacillin-tazobactam during hospital stay was effective, but there were relapses shortly after discharge with oral antibiotics.

We believe the incidence of *P. otitidis* infections in patients with chronic lung diseases may be largely underestimated due to its similarity to *P. aeruginosa*, which can lead to the bacterium’s misclassification and an underestimation of its clinical impact.

## Data Availability

Not applicable.

## Abbreviations

BPM: Beats per minute
P: Pseudomonas
COPD: Chronic obstructive pulmonary disease
Cpm: Cycles per minute
CRP: C-reactive protein
CT: Computed tomography
MALDI-TOF MS: Matrix-assisted laser desorption/ionisation time-of-flight mass spectrometry
